# Identification of Reference and Biomarker Proteins in *Chlamydomonas reinhardtii* Cultured under Different Stress Conditions

**DOI:** 10.3390/ijms18081822

**Published:** 2017-08-22

**Authors:** Jianan Shi, Teng Huang, Shuaijie Chai, Yalu Guo, Jian Wei, Shijuan Dou, Liyun Li, Guozhen Liu

**Affiliations:** Institute of Bioenergy, College of Life Sciences, Hebei Agricultural University, Baoding 071001, Hebei, China; shijianan_mbb@126.com (J.S.); huangteng_mbb@126.com (T.H.); chaishuaijie_mbb@126.com (S.C.); Guoyalu_mbb@126.com (Y.G.); weijian_mbb@126.com (J.W.); dsj75@126.com (S.D.); liliyun@hebau.edu.cn (L.L.)

**Keywords:** *Chlamydomonas reinhardtii*, abiotic stress, reference protein, biomarker, microalgae, antibody, western blotting

## Abstract

Reference proteins and biomarkers are important for the quantitative evaluation of protein abundance. *Chlamydomonas* *reinhardtii* was grown under five stress conditions (dark, cold, heat, salt, and glucose supplementation), and the OD_750_ and total protein contents were evaluated on days 0, 1, 2, 4, and 6 of culture. Antibodies for 20 candidate proteins were generated, and the protein expression patterns were examined by western blotting. Reference protein(s) for each treatment were identified by calculating the Pearson’s correlation coefficient (PCC) between target protein abundance and total protein content. Histone H3, beta tubulin 1 (TUB-1), ribulose-1,5-bisphosphate carboxylase/oxygenase large subunit (RBCL), and mitochondrial F1F0 ATP synthase subunit 6 (ATPs-6) were the top reference proteins, because they were expressed stably under multiple stress conditions. The average relative-fold change (ARF) value of each protein was calculated to identify biomarkers. Heat shock protein 90B (HSP90B), flagellar associated protein (FAP127) and ATP synthase CF0 A subunit (ATPs-A) were suitable biomarkers for multiple treatments, while receptor of activated protein kinase C1 (RCK1), biotin carboxylase (BCR1), mitochondrial phosphate carrier protein (MPC1), and rubisco large subunit *N*-methyltransferase (RMT1) were suitable biomarkers for the dark, cold, heat, and glucose treatments, respectively.

## 1. Introduction

Microalgae are photosynthetic microorganisms that are used in a wide variety of applications. Due to their relatively high lipid/biogas productivity, microalgae are promising for biofuel production [1,2]. They can also be used to capture CO_2_ and remove nutrients from wastewater, thus playing an important role in wastewater treatment [3]. Microalgae can biosynthesize various pigments, antioxidants, β-carotenes, proteins, and vitamins, which can be used to produce high-value products. They can be grown on non-arable land and require less land than terrestrial crops; thus, they neither compromise food production nor compete with agriculture [4]. Although these microorganisms can grow in a wide variety of environmental conditions, microalgae production still faces significant problems related to stress during the culture process.

Since the whole genome sequence [5] and mutant libraries [6] have become available, *Chlamydomonas reinhardtii* has become a promising model organism in molecular biology research. Currently, it is widely used in research on abiotic stress responses, biofuels, biosynthesis, photosynthesis, respiration, and the circadian clock [7,8]. Understanding changes in protein abundance under different stresses is important to clarify gene function.

Abiotic stress may affect the growth of *C. reinhardtii* in different ways. Hemshmeier study [9] indicates that nearly 1400 genes were differentially expressed, triacylglycerol accumulation increased, and the nitric oxide-dependent pathway was activated under dark and hypoxic conditions. It was also reported that dark treatment made *C. reinhardtii* cells more sensitive to ultraviolet radiation [10]. Cold stress was shown to promote sugar and starch accumulation in cells, change in membrane lipid composition, and decrease of mobility [11]. Cold stress was also shown to down-regulate the biomass and lipid content in *C. reinhardtii* cells, however, biomass accumulation in the arctic alga *Chlamydomonas* sp. KNM0029C remained stable when it was cultured at low temperature [12]. The effects of global warming on crop yield have led to research on the heat tolerance mechanisms of plants. The heat stress response (HSR) occurs in *C. reinhardtii* cells at 39–41 °C [13], and heat shock proteins (HSPs) are up-regulated as biomarkers of the HSR [14]. Heat shock for 3–24 h resulted in increased contents of molecular chaperones such as CPN, CGE, and BIP, while the large subunit (LSU) and small subunit (SSU) of ribulose-1,5-bisphosphate carboxylase (RubisCO) were downregulated [15,16]. The heat tolerance can be enhanced by overexpression of endogenous photosynthetic FDX gene, *PETF* [17]. Under heat stress, Légeret et al. unveiled a direct route for the conversion of membrane lipids into storage lipids based on lipidomic and transcriptomic analyses of *C. reinhardtii* [18]. Salt stress is one of the most common factors limiting crop yield. An LC-MS/MS analysis of *C. reinhardtii* under salinity stress revealed that 18 proteins were down-regulated and 99 proteins were up-regulated [19]. *C. reinhardtii* cells cannot utilize glucose as a carbon resource [20], however, it was reported that an infinitesimal amount of glucose can be transported into *Chlamydomonas* cells [21]. Protein is the executor for nearly all biological processes in living creatures, western blot is an exceptional analytical technique used for the detection of protein abundance. Reference proteins provide the basis for quantitative comparisons of protein abundance. Various *C. reinhardtii* proteins were used as the loading quantification markers in WB analysis, the β-subunit of the CF0F1 ATPase (ATPs-β) was used as a loading control to detect the accumulation of light-harvesting complex stress-related (LHCSR) proteins in wild-type and mutant strains at different time points during high light exposure [22]. The mRNA encoding receptor of activated protein kinase C1 (RCK1) (*Chlamydomonas* beta subunit-like polypeptide; *Cblp*) was constitutively expressed during the cell cycle and flagella regeneration [23] and, therefore, was used as a loading control in Northern blot analyses [24]. Its homolog in rice (rice protein containing WD-40 repeat, RWD) was also found to be constitutively expressed [25]. Photosystem II protein D1 (D1) is one of the core proteins of photosystem II and is commonly used to normalize the amounts of proteins in samples, especially in high light/low light (HL/LL) treatments [26]. When *Chlamydomonas* proteins were detected in wild-type, *cpld38*, and *cpld38-CPLD38* rescued strains by western blotting, the abundance of three common reference proteins (ATPs-β, ribulose-1,5-bisphosphate carboxylase/oxygenase large subunit (RBCL), LHCBM1) changed, while the abundance of α-tubulin remained stable. Thus, α-tubulin was used as the loading control [27]. In wild-type and the *ccm*1 and *Ccm*1 mutants, the LSU of RubisCO was expressed constitutively and was, therefore, used as the reference protein [24]. In another study, Histone H3 served as the loading control to check the abundance of the LSU and SSU of RubisCO in different samples [28].

Biomarkers are not only used as a reference for quantitative protein analysis, but also as an indicator of a stress response or the extent of the stress. Up-regulation of *HSP* genes are commonly accepted as an indicator of the HSR. In *Fucus serratus* and *Lemna minor*, HSP70 was considered to be a suitable stress-specific biomarker under short-term heat, osmotic, and cadmium stresses [29]. In *C. reinhardtii*, HSP70B was used as an early marker for induced oxidative stress [30], and Rubisco can be used as an indicator for the accumulation of Calvin-Benson cycle enzymes [31].

Organelle biomarkers can play important roles in localizing target proteins within the cell. Ferritin1 and Ferritin2 were located in the chloroplast by using keto-acid acid isomeroreductase (KARI) as a chloroplast biomarker [32]. Cytochrome c oxidase subunit II (COX2b) was used as a biomarker for mitochondria and flagellar outer dynein arm intermediate chain 2 (IC2) was used as a biomarker for flagella [33,34]. The identification, validation, and application of biomarkers are essential for functional research on proteins.

Knepper et al. [35] described a targeted proteomic approach using antibodies, which was applied to select reference proteins and biomarkers in human and model animals [36,37]. Previously, we identified a HSP and elongation factor 1α (eEF-1α) as reference proteins in rice [38], and the expression of pathogenesis-related (PR) proteins was investigated as biomarkers of the rice defense response against *Xathomonas oryzae* pv. *oryzae* [39,40].

Reference and biomarker proteins are key elements for protein analysis. However, research to systematically identify and validate reference and biomarker proteins under abiotic stresses in microalgae is still limited. In this study, *C. reinhardtii* cells were cultivated under control (CK) and five different stress conditions. We monitored cell density and total protein content, and evaluated the expression patterns of 20 candidate proteins at different time points by Western blotting. The Pearson’s correlation coefficient (PCC) between each specific protein and cell density/total protein was calculated to identify the most suitable reference proteins. Also, the average relative-fold change (ARF) value was calculated for each protein under each of the stress conditions. Biomarkers were identified as those with the most distinct ARF compared with the CK in multiple or specific treatments. The results not only characterized the expression of candidate proteins under stress conditions, but also identified suitable reference and biomarkers that will be useful to the research community working on microalgae proteomics.

## 2. Results and Discussion

### 2.1. Growth of C. reinhardtii Cells under Stress

**Figure 1:**
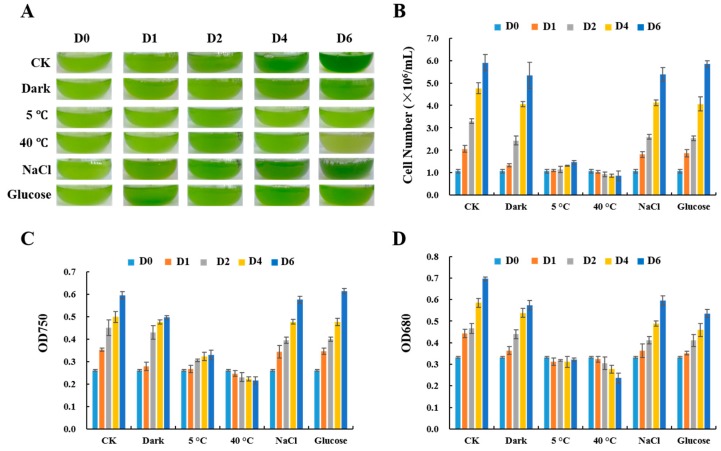
Culture appearance and cell density of *Chlamydomonas reinhardtii* grown under abiotic stresses. (**A**) Photos taken of *C. reinhardtii* at different time points during culture: control (CK), dark, cold (5 °C), heat (40 °C), NaCl (100 mM), and glucose (1% *w*/*v*); (**B**) Cell number was determined using a hemocytometer under a microscope; (**C**,**D**) Optical density of the culture at 750 nm and 680 nm in various treatments. Data are the average values from three independent experiments.

The morphology appearance of *C. reinhardtii* cells cultured under different stress conditions is shown in Figure 1A, and the cell number, OD_750_, and OD_680_ growth curves are shown in Figure 1B–D, respectively.

In CK, the culture color changed from light to dark green during the six-day culture period, and the number of cells increased for about five times. Both the OD_750_ and OD_680_ value increased and showed interdependent curves, indicating a normal propagation of *C. reinhardtii* cells. Under dark conditions, the cell number and OD_750_ were lower than that in CK, and the color was lighter, corresponding to the decrease of OD_680_, support that chlorophyll synthesis was down-regulated in the dark. Cell growth was very slow under cold stress. The final cell number and OD_750_ were increased slightly than that on day 0, while OD_680_ remained stable. The cells turned light yellow under heat stress, and the cell number, OD_750_, and OD_680_ decreased, even lower than their initial value, indicating that high temperature not only inhibited cell growth, but also led to cell death. Under NaCl treatments, cell number, OD_750_, and OD_680_ were slightly lower than that in the control. In glucose supplementation treatments, cell number and OD_750_ were higher than that in the control, while OD_680_ was lower than the control.

In summary, among the five stresses tested, heat stress most strongly inhibited the growth and function of *C. reinhardtii* cells, as indicated by the prevailing decreases in cell number, OD_750_, and OD_680_ in heat-treated cells. Dark, cold, salt, and glucose treatments also affected the growth of *C. reinhardtii* cells, but to a lesser extent.

### 2.2. Total Protein Alteration of C. reinhardtii Cells under Stress

**Figure 2:**
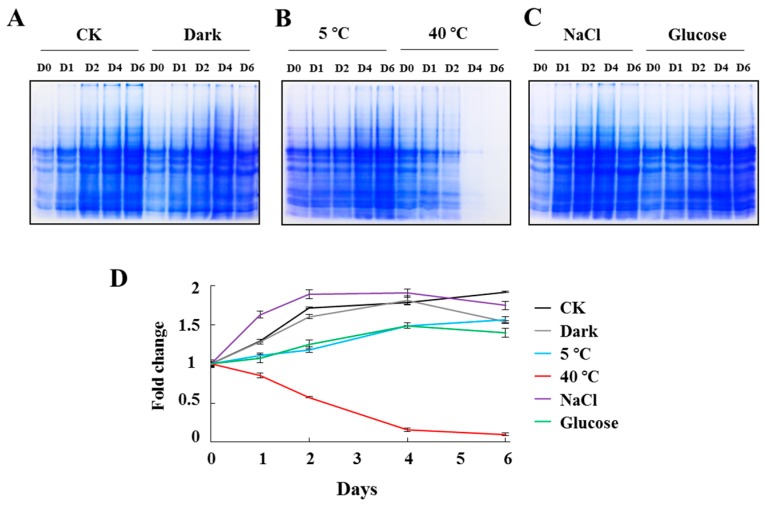
Total protein contents of *Chlamydomonas reinhardtii* cells cultured under various abiotic stresses. *C. reinhardtii* cells were collected by centrifugation at indicated time points, and total proteins were extracted and separated by sodium dodecyl sulfate polyacrylamide gel electrophoresis (SDS-PAGE). (**A**–**C**). Coomassie Brilliant Blue-stained gels. (**D**). Signals extracted from A–C by Lane 1D software. To normalize data collected from different gels, value at day 0 was set to one and fold changes at other time points were calculated. Data are average values from three independent experiments. Cells were grown without stress (CK, control) or in the dark, cold (5 °C), heat (40 °C), or with NaCl or glucose supplementation. Samples were collected on days 0, 1, 2, 4, and 6 of culture.

Total proteins were extracted at different time points and separated by SDS-PAGE, and then the CBB signals (Figure 2A–C) were quantified by Lane 1D software (Figure 2D). Total proteins were also quantified by BCA analysis, which produced results consistent with those in Figure 2D.

In CK, the total protein content doubled along with the increase of cell number during the six-day culture. The total protein content in dark-treated *C. reinhardtii* cells increased as the cell density increased at the early stage (day 0–4), but decreased at day 6, suggesting that protein synthesis slowed down under prolonged dark stress. Although cell growth was very slow under cold stress, the total protein had increased about 50% at day 6, implying that cold stress may promote the biosynthesis of protein [11]. Under heat stress, *C. reinhardtii* cells resulted in extensive cell death and protein degradation. The amount of protein at day 4 and 6 were barely detectable using CBB. It was reported that one day of heat shock (42 °C) led to a 1.58-times increase in protein concentration per cell [16]. Cells grown with NaCl can promote the accumulation of proteins, and the protein content peaked at day 2. This result suggested that a high ion concentration stimulated protein synthesis. In glucose supplementation experiment, the total protein content on day 6 was 1.5-times than that on day 0, but lower than that in CK, indicated that glucose in the medium may inhibit protein synthesis.

### 2.3. Recombinant Protein Expression, Antibody Generation, and Specificity Validation

**Figure 3:**
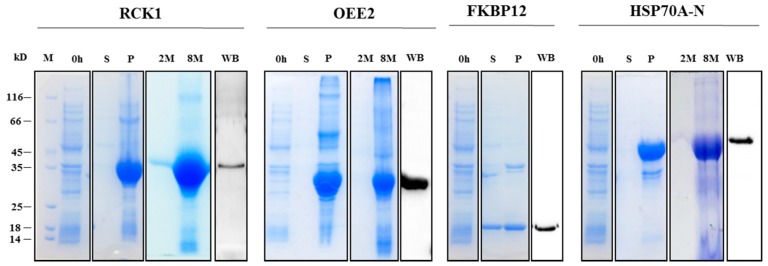
Recombinant protein expression and antibody verification. Candidate genes (*RCK1*, *OEE2*, *FKBP12*, and *HSP70A*-N terminal sequence) were cloned into expression vector pET30a and transformed into *Escherichia coli*. Expression of recombinant proteins was induced by adding isopropyl β-d-1-thiogalactopyranoside (IPTG) to lysogeny broth (LB) medium. *E. coli* cells were collected by centrifugation and total proteins were extracted after sonication. Supernatant and pellet samples were separated by centrifugation. Recombinant proteins in pellet form were washed in 2 M urea and then dissolved in 8 M urea. SDS-PAGE-separated proteins were visualized by Coomassie Blue staining. Dissolved recombinant protein was used to generate polyclonal antibodies. To verify the specificity of antibodies, Western blotting analysis was carried out against dissolved proteins. M: molecular weight marker; 0 h: samples collected at 0 h; S: supernatant samples collected at 3 h after IPTG induction. P: pellet samples collected at 3 h after IPTG induction; 2 M: pellet samples washed with 2 M urea; 8 M: pellet samples dissolved in 8 M urea; WB: SDS-PAGE-separated proteins were immobilized on polyvinylidene difluoride (PVDF) membrane and detected with antibodies against recombinant proteins.

**Figure 4:**
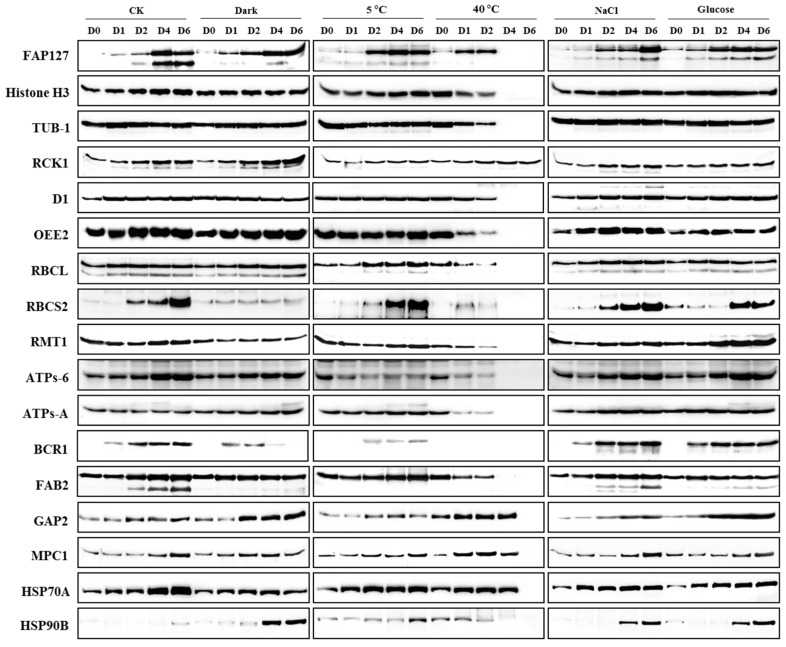
Western blot detection of candidate proteins in *Chlamydomonas reinhardtii* cells under abiotic stresses at different time points. *C. reinhardtii* cells cultured under different abiotic stress conditions were collected at five time points. Total proteins were extracted and separated by SDS-PAGE. PVDF-membrane-immobilized proteins were detected by antibodies against 20 candidate proteins. CK, Dark, 5 °C, 40 °C, NaCl, and Glucose are treatments for *C. reinhardtii* cells. D0, D1, D2, D4, and D6 are the time points (days) of sample collection.

Four recombinant proteins (RCK1, OEE2, FKBP12, and HSP70A-N) were expressed. Among them, FKBP12 was detectable in both the supernatant and inclusion bodies, while the other three were detectable only in the pellet fraction. The purified proteins were used as antigens to generate polyclonal antibodies. Western blotting analysis showed that the antibodies could recognize recombinant antigens (Figure 3). Dot-blot assays were performed to validate the specificity of 16 peptide-derived antibodies; 13 antibodies specifically bound to the corresponding antigens, while three antibodies (against IC2, beta tubulin 1 (TUB-1), and mitochondrial phosphate carrier protein (MPC1)) not only bound to their antigen peptide, but also cross-reacted with the glyceraldehyde 3-phosphate dehydrogenase (GAP2) peptide. However, these three antibodies did not detect the native GAP2 protein in *C. reinhardtii* cells (Figure 4), indicating that the cross-reactivity with GAP2 peptides in vitro did not interfere with Western blotting analyses of native proteins. Together, the Western blotting and dot-blot assays provided evidence for the specificity of the antibodies.

### 2.4. Expression Profiling of Candidate Proteins

Expression profiling of the 20 candidate proteins was determined by Western blotting using 21 antibodies to analyze samples collected at five time points from six treatments (21 antibodies × 6 treatments × 5 time points = 630 Western blot lanes). Among them, three peptide-derived antibodies (IC2, FKBP12, and ATPs-β) and the antibody against the HSP70A-N recombinant protein did not detect any signals (data not shown). The Western blotting results generated for 17 proteins in six treatments are shown in Figure 4. Out of 17 proteins, 14 showed a single band while the other three produced two bands in parallel, possibly due to post-translational modifications. In day 0 samples, 13 proteins showed clear signals, while four proteins showed background signals. However, clear signals were detectable in the treated samples. In the CK samples, most proteins showed an up-regulated pattern as the *C. reinhardtii* cells proliferated. The most remarkable phenomena were observed in heat-treated samples; that is, RCK1, GAP2, MPC1, and HSP70A showed thermo-stable expression patterns as their signals at day 4 were almost the same as those at day 2, even though the total protein level at that time was extremely low (Figure 2). The RCK1 signal remained stable at day 6 of heat stress. Heat stress resulted in cell death due to damage to protein folding, complex assembly, homeostasis, and membrane fluidity [14]. Almost all proteins were degraded when the cells were incubated at 40 °C for 4–6 days (Figure 2). The identification of heat-tolerance proteins may have important practical applications. To show the data more intuitively, signals from the Western blots were extracted and normalized to a value of 1 on day 0, and then line charts were drawn to show fold changes. This systematic western blotting analysis provided valuable data for the selection of reference and biomarker proteins.

### 2.5. Identification of Reference Proteins under Abiotic Stresses

Reference proteins are those that are stably expressed or proportionally expressed as the biomass changes under specific conditions. To identify reference proteins, PCC analyses were carried out between normalized intensities of candidate proteins at different times and OD_750_ or total protein content. The PCCs between OD_750_ and total protein content for each treatment were also calculated (Table 1). As shown in Table 1, the PCC between total protein content and OD_750_ was 0.988 in the CK (*p* < 0.01), and 0.935 and 0.890 in the glucose and 40 °C treatments, respectively (*p* < 0.05). The PCCs showed no significant correlation for the other three treatments (dark, 5 °C, and salt).

In the CK, the expression of 13 out of 17 proteins was significantly correlated with OD_750_ and the expression of 11 of those 13 proteins was correlated with total protein content. In the other treatments, the number of proteins whose expression was correlated with OD_750_ and total protein content were as follows: eight and six, respectively, in the dark treatment, with four overlapping; one and 10, respectively, in the 5 °C treatment, with no overlap; five and nine, respectively, in the 40 °C treatment, with five overlapping; 13 and five, respectively, in the salt treatment, with four overlapping; and 12 and seven, respectively, in the glucose treatment, with seven overlapping. Based on these results, we conducted that the higher the correlation between OD_750_ and total protein in a treatment, the more proteins were significantly correlated with either OD_750_ or total protein content.

Almost all candidate proteins showed a correlation with either OD_750_ or total protein content in at least one treatment, which provided multiple options for the selection of reference proteins. However, it is better if the expression of a reference protein is correlated with changes in protein content. Thus, the reference proteins were selected based on the number of treatments in which their abundance was correlated with total protein content. The abundance of BRCL and mitochondrial F1F0 ATP synthase subunit 6 (ATPs-6) was significantly correlated with total protein content in five out of six treatments, and the abundance of Histone H3 and TUB-1 was significantly correlated with total protein content in four treatments. These four proteins were selected as the first batch of reference proteins. We note, however, that these four reference proteins may not be suitable in other conditions, and a different reference protein may be selected based on specific experiments.

### 2.6. Identification of Biomarkers under Abiotic Stresses

**Figure 5:**
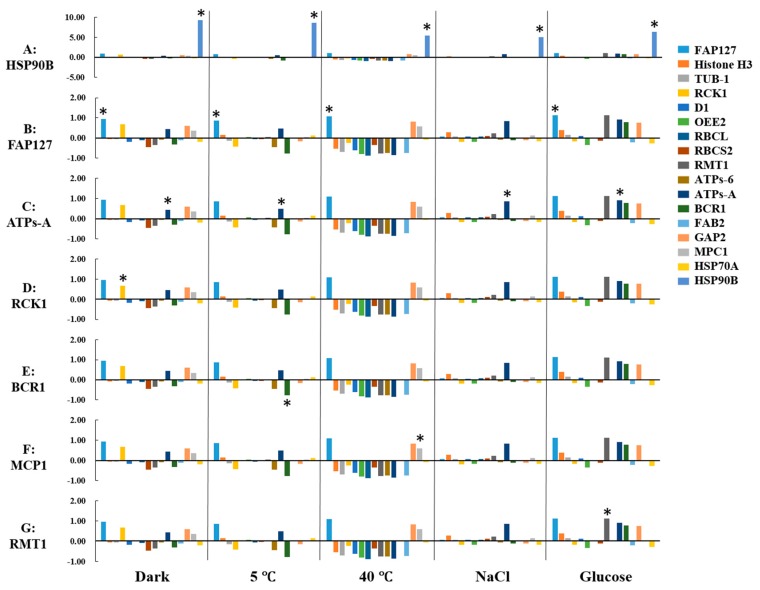
Average relative fold change (ARF) of candidate proteins and selected biomarkers. ARFs calculated for candidate proteins are illustrated by different colored bars (see key). For each protein, ARF was calculated as follows: ∑1, 2, 4, 6(I(Treatment) − I(CK))/I(CK))/4, where 1, 2, 4, and 6 are days 1, 2, 4, and 6, respectively, I(treatment) is normalized intensity of Western blot for specific treatment at n (1, 2, 4, or 6) time points, and I(CK) is the normalized intensity of the Western blot for the control (CK). “*” indicates the recommended biomarker with highest ARF under multiple or specific treatments. (**A**) ARF value of HSP90B was highlighted with “*” indicating it is a wide-range biomarker for five treatments. HSP90B data were excluded in (**B**–**G**) and “*” designated the recommended biomarkers respectively. (**B**) highlights the ARF of FAP127. (**C**) highlights the ARF of ATPs-A. RCK1 is highlighted with “*” in (**D**), BCR1 in (**E**), MPC1 in (**F**) and RMT1 in (**G**).

Biomarkers are the proteins that are differentially expressed in treatments as compared with the control. Their unique expression may indicate whether a certain stress occurred. To identify biomarkers, the ARF was calculated for each protein under the five treatments (Figure 5A). Heat shock protein 90B (HSP90B) showed the highest ARF among five treatments, indicating that this protein was a wide-range biomarker suitable for these five treatments. The high ARF value of HSP90B masked the ARFs of other proteins; therefore, to show the ARFs of the other proteins clearly, HSP90B data were excluded in Figure 5B–G. Figure 5B highlights the ARF of flagellar associated protein (FAP127), which was differentially expressed in the dark, 5 °C, 40 °C, and glucose treatments, but not in the salt treatment. Figure 5C highlights the ARF of ATP synthase CF0 A subunit (ATPs-A), which was differentially expressed in the dark, 5 °C, salt, and glucose treatments, but not in the 40 °C treatment. RCK1 was specifically expressed in the dark treatment (Figure 5D); biotin carboxylase (BCR1) in the 5 °C treatment (Figure 5E), MPC1 in the 40 °C treatment (Figure 5F) and rubisco large subunit *N*-methyltransferase (RMT1) in the glucose treatment (Figure 5G). In conclusion, HSP90B, FAP127, and ATPs-A were suitable biomarkers for multiple treatments, while RCK1, BCR1, mitochondrial phosphate carrier protein (MPC1), and RMT1 were suitable biomarkers for the dark, cold, heat, and glucose treatments, respectively.

## 3. Materials and Methods

### 3.1. C. reinhardtii Strain and Culture

*C. reinhardtii* CC-124 was obtained from the Chlamydomonas Resource Center [41]. A single clone was obtained on solid tris-acetate-phosphate (TAP) medium (Gorman and Levine 1965) and then grown in TAP liquid medium at 25 °C under a 12 h dark/12 h light (100 µmol/(m^2^·s)) photoperiod on a rotary shaker (120 rpm) for two days until it reached the logarithmic phase (OD_750_ ≈ 0.3). The cells were then collected for further culture under abiotic stress conditions. All reagents were purchased from the Sangon Biotech Co., Ltd. (Shanghai, China).

### 3.2. Abiotic Stress Treatments

For the control (CK), *C. reinhardtii* cells were grown at 25 °C under a 12 h dark/12 h light photoperiod. For the dark treatment, Erlenmeyer flasks were fully wrapped in a black cloth. For the cold and heat stress treatments, the flasks were cultured in illuminated incubators set at 5 °C and 40 °C, respectively. For the salt and glucose treatments, the cells were grown in medium with a final concentration of 100 mM sodium chloride and 1% (*w*/*v*) glucose, respectively. Stock solution of 40% glucose, prepared by filtering through 0.2 micron filter, was supplemented to autoclaved medium. *C. reinhardtii* cells were collected five times (on days 0, 1, 2, 4, and 6) for analysis.

### 3.3. Detection of Cell Density and Cell Number

The optical density at 750 nm (OD_750_) and 680 nm (OD_680_) of the culture was measured using a spectrophotometer (WFJ 7200, Unico, Shanghai, China). Cell number was counted under a microscope using hemocytometer. Each experiment was repeated at least three times and curves for cell density and total protein were drawn.

### 3.4. Candidate Proteins

Twenty selected candidate proteins and related information are listed in Table 2, including Au10_locus ID, predicted subcellular position, annotation, and predicted molecular weight, type of antigen used, peptide sequence, and related references.

### 3.5. Recombinant Protein Expression and Antibody Generation

Total RNA was extracted from *C. reinhardtii* cells and reverse transcribed into cDNA for use as PCR templates using a plant total RNA isolation kit and reverse transcription kit (Sangon Biotech Co., Ltd., Shanghai, China), according to the manufacturer’s instructions. Full-length cDNAs of RCK1, oxygen-evolving enhancer protein 2 of photosystem II (OEE2), and peptidyl-prolyl cis-trans isomerase FKBP-type (FKBP12) and a 1200-nt fragment from the 5′-terminal of heat shock protein 70A (HSP70A) were amplified and then each cloned into an expression vector as reported previously [38]. Proteins enriched in inclusion bodies were washed with 2 M urea and dissolved in 8 M urea. The primers and restriction endonucleases used are listed in supplemental Table 2. The unique epitopes of the other 16 candidate proteins in *C. reinhardtii* were predicted by PepDesign software [49], and the peptides with the top scores were chosen. The synthesized peptides were used as antigens to inject rabbits. Peptide synthesis and antibody generation were carried out by the Beijing Protein Innovation Co., Ltd. (Beijing, China).

### 3.6. C. reinhardtii Protein Extraction and Quantification

Total protein was extracted from *C. reinhardtii* cells as described previously with minor modifications [50]. Briefly, 30 mL *C. reinhardtii* culture was centrifuged at 10,000× *g* for 1 min, and the cell pellet was re-suspended in 1 mL extraction buffer (60 mM DTT, 60 mM Na_2_CO_3_, 2% *w*/*v* SDS, 12% *w*/*v* sucrose, 100 µL zirconium beads), shaken for 20 min at room temperature, centrifuged at 10,000× *g* at 4 °C for 10 min, and the supernatant was collected. For Western blotting, 200 µL 5× loading buffer (250 mM Tris-HCl, (pH 6.8), 10% SDS (*w/v*), 0.5% bromophenol blue (*w/v*), 50% glycerol (*v/v*), 5% β-mercaptoethanol) was added to 800 µL protein solution. The mixture was vortexed briefly, then heated in a boiling water bath for 10 min, and finally stored at −20 °C until use. For absolute quantification, bovine serum albumin (BSA) was used to construct a standard curve. To measure the protein content in samples, 10 µL protein solution (without loading buffer) was mixed with 50 µL cold acetone (−20 °C). The mixture was kept at −20 °C for 2 h and then centrifuged for 15 min at 5000× *g* at 4 °C. The pellet was washed twice with acetone and dried in a fume hood, and then dissolved in 10 µL ddH_2_O. The protein concentration was determined using a Fluorescent BioSpectrometer (Eppendorf, Hamburg, Germany) according to the manufacturer’s instructions. For relative quantification, protein samples were separated using 10% sodium dodecyl sulfate polyacrylamide gel electrophoresis (SDS-PAGE) and stained with Coomassie Brilliant Blue R-250 (Sangon Biotech Co., Ltd., Shanghai, China). The average and standard derivation were calculated based on the extracted signals.

### 3.7. Western Blot Analyses

Western blot analyses were carried out as described previously [38]. Briefly, total proteins were separated by SDS-PAGE and electro-transferred to a polyvinylidene difluoride (PVDF) membrane (Millipore Corporation, Bedford, MA, USA), then blocked with 5% non-fat milk in TTBS (0.2 M Tris-HCl (pH 7.6), 1.37 M NaCl, 0.1% *v*/*v* Tween-20) solution for 1 h, incubated for 3 h with primary antibody diluted 1:1000 in TTBS solution and for 1 h with a horseradish peroxidase-conjugated goat anti-rabbit secondary antibody (Beijing Protein Innovation Co., Ltd., Beijing, China) diluted 1:15,000 in tris-buffered saline and polysorbate 20 (TTBS) solution. The blot was developed with an eECL Western Blot kit (Cwbiotech Co., Ltd., Beijing, China) and the signals were captured and extracted by a Mini Chemiluminescent Imaging and Lane 1D Analysis System (Sage Creation Science Co., Ltd., Beijing, China).

### 3.8. Pearson’s Correlation Coefficient (PCC) Analysis

We used IBM SPSS v. 24 (IBM Corp., Armonk, NY, USA) to calculate Pearson’s correlation coefficient (PCC) between the abundance of each target protein and total protein/OD_750_. The normalized Western blot intensity indicated the abundance of the target protein. To normalize Western blot intensity, the intensity at day 0 from different Western blots was averaged and set to 1, and the intensities at days 1, 2, 4, and 6 were calculated accordingly.

### 3.9. Average Relative Fold Change (ARF) Calculation and Biomarker Selection

The ARF for each protein was calculated as follows:

ARF = (∑1, 2, 4, 6(I(Treatment) − I(CK))/I(CK))/4(1)

where 1, 2, 4, and 6 are days 1, 2, 4, and 6, respectively, I(treatment) is the normalized intensity of the Western blot for a specific treatment at n (1, 2, 4, or 6 days), and I(CK) is the normalized intensity of the Western blot in the CK. The fold change at a specific time point was calculated by I(n)/I0, where I(n) is the intensity of the Western blot at n (1, 2, 4, or 6 days), and I0 is the intensity of the Western blot at day 0.

## 4. Conclusions

*C. reinhardtii* was cultivated under five stress conditions: dark, cold, heat, salt, and glucose supplementation. Based on the OD_750_ and protein contents of cells at days 0, 1, 2, 4, and 6, and the expression patterns of 20 proteins detected by western blotting, we identified reference and biomarker proteins. Reference protein(s) for each treatment were identified by calculating the Pearson’s correlation coefficient (PCC) between target protein abundance and total protein content. Histone H3, TUB-1, RBCL, and ATPs-6 were the top reference proteins, because they were expressed stably under multiple stress conditions. The average relative-fold change (ARF) value of each protein was calculated to identify biomarkers. HSP90B, FAP127 and ATPs-A were suitable biomarkers for multiple treatments, while RCK1, BCR1, MPC1, and RMT1 were suitable biomarkers for the dark, cold, heat, and glucose treatments, respectively. These results and antibody resources will be useful for further proteomics research on *C. reinhardtii*.

## Figures and Tables

**Table 1:** Correlation analyses between abundance of candidate proteins and OD_750_/total protein content of *C. reinhardtii* cells. IBM SPSS statistics v. 24 (IBM Corp. Armonk, NY, USA) was used to calculate Pearson’s correlation coefficient (PCC) between normalized Western blot intensity of target protein and total protein/OD_750_. To normalize Western blot intensity, intensity at day 0 from different blots was averaged and set to 1, and intensity at days 1, 2, 4, and 6 was calculated accordingly. “Protein with *” indicates number of proteins with significant (* or **) correlation with OD_750_ or total protein content. Number in brackets indicates number of overlapping proteins. TP: total protein content; “TP with *”: number of experiments with significant (* or **) correlation between total protein content and specific protein expression. **: significant at the 0.01 level; *: significant at the 0.05 level; -: not available as BCR1 protein was not detectable during heat treatment.

	CK		Dark		5 °C		40 °C		NaCl		Glucose		
	OD_750_	TP	OD_750_	TP	OD_750_	TP	OD_750_	TP	OD_750_	TP	OD_750_	TP	TP with *
**OD_750_**	1.000	0.988 **	1.000	0.781	1.000	0.626	1.000	0.890 *	1.000	0.844	1.000	0.935 *	
**FAP127**	0.913 *	0.862	0.949 *	0.921 *	0.634	0.905 *	0.133	0.434	0.948 *	0.633	0.999 **	0.933 *	3
**Histone H3**	0.957 *	0.950 *	0.685	0.827	0.697	0.937 *	0.722	0.916 *	0.963 **	0.949 *	0.879 *	0.713	4
**TUB-1**	0.953 *	0.949 *	0.787	0.769	0.480	0.956 *	0.819	0.982 **	0.903 *	0.986 **	0.930 *	0.765	4
**RCK1**	0.945 *	0.944 *	0.996 **	0.808	0.491	0.868	−0.870	−0.849	0.948 *	0.769	0.994 **	0.899 *	2
**D1**	0.977 **	0.946 *	−0.541	−0.336	0.208	0.650	0.812	0.974 **	0.997 **	0.804	0.968 **	0.865	2
**OEE2**	0.974 **	0.969 **	0.937 *	0.866	0.439	0.742	0.737	0.952 *	0.944 *	0.963 **	0.984 **	0.868	3
**RBCL**	0.929 *	0.895 *	0.862	0.919 *	0.800	0.881 *	0.914 *	0.921 *	0.938 *	0.771	0.984 **	0.879 *	5
**RBCS2**	0.883 *	0.862	0.856	0.896 *	0.747	0.978 **	0.449	0.778	0.914 *	0.615	0.795	0.876	2
**RMT1**	0.819	0.787	−0.678	−0.491	0.912 *	0.799	0.901 *	0.954 *	0.938 *	0.637	0.975 **	0.959 **	2
**ATPs-6**	0.939 *	0.918 *	0.920 *	0.958 *	−0.666	−0.944 *	0.920 *	0.996 **	0.959 **	0.782	0.962 **	0.994 **	5
**ATPs-A**	0.384	0.279	0.986 **	0.818	0.523	0.917 *	0.964 **	0.889 *	0.932 *	0.919 *	0.778	0.592	3
**BCR1**	0.961 **	0.982 **	−0.125	−0.002	0.745	0.836	-	-	0.884 *	0.681	0.929 *	0.790	1
**FAB2**	0.915 *	0.914 *	−0.286	0.207	0.668	0.973 **	0.958 *	0.982 **	0.915 *	0.774	−0.815	−0.851	3
**GAP2**	0.903 *	0.957 *	0.893 *	0.824	0.701	0.991 **	−0.275	0.071	0.798	0.577	0.973 **	0.963 **	3
**MPC1**	0.762	0.708	0.930 *	0.944 *	0.854	0.899 *	0.000	0.315	0.831	0.431	0.804	0.836	2
**HSP70A**	0.900 *	0.848	0.951 *	0.920 *	0.515	0.764	−0.045	0.287	0.844	0.922 *	0.985 **	0.901 *	3
**HSP90B**	0.531	0.457	0.827	0.668	0.860	0.838	0.149	0.361	0.778	0.427	0.770	0.804	0
Protein with *	13(11)11		8(4)6		1(0)10		5(5)9		13(4)5		12(7)7		

**Table 2:** List of candidate genes and related information.

No.	Gene Name	Au10_Locus/ID	Category	Subcellular Localization	Annotation	MW (kD)	Antigen Sequence	References
1	FAP127	Cre17.g737100	Structure	Motile cilium	Flagellar associated protein	51	YSEGLQEDKKIRN	
2	Histone H3	Cre06.g268350	Structure	Nucleus	Histone H3	15	TELLIRKLPFQRLV	[28]
3	IC2	Cre12.g506000	Structure	Flagella	Flagellar outer dynein arm Intermediate chain 2	64	WDLRKMNECVENMPL	[33,42]
4	TUB-1	Cre12.g542250	Structure	Cytoskeleton	Beta tubulin 1	50	QYQDATADEEGEYEDEEQQ	
5	FKBP12	Cre13.g586300	Signaling	Endoplasmic reticulum	Peptidyl-prolyl cis-trans isomerase FKBP-type	12	Expressed protein (Full length)	[43]
6	RCK1	Cre06.g278222	Signaling	Whole cell	Receptor of activated protein kinase C1	35	Expressed protein (Full length)	[44]
7	D1	NP_958377.1	Photosynthesis	Chloroplast	Photosystem II protein D1	39	IRETTENESANE	[26]
8	OEE2	Cre12.g550850	Photosynthesis	Chloroplast	Oxygen-evolving enhancer protein 2 of photosystem II	30	Expressed protein (Full length)	[45]
9	RBCL	NP_958405.1	Photosynthesis	Chloroplast	Ribulose-1,5-bisphosphate carboxylase/oxygenase large subunit (chloroplast)	53	MRDDYVEKDRSR	[46]
10	RBCS2	Cre02.g120150	Photosynthesis	Chloroplast	Ribulose-15-bisphosphate carboxylase/oxygenase small subunit 2	21	QRPKSARDWQPA	
11	RMT1	Cre16.g661350	Photosynthesis	Chloroplast	Rubisco large subunit *N*-methyltransferase	52	LRPSMTYSITPDQQ	
12	ATPs-6	Cre01.g018800	Metabolism	Mitochondria	Mitochondrial F1F0 ATP synthase subunit 6	35	NMMAGHSSVKILSG	
13	ATPs-A	ACS16334	Metabolism	Mitochondria	ATP synthase CF0 A subunit, partial (chloroplast)	13	AGLSKKGLSYFEKY	
14	ATPs-β	Cre17.g698000	Metabolism	Mitochondria	ATP synthase CF1 beta subunit	53	FAGVGERTREGNDL	[22]
15	BCR1	Cre08.g359350	Metabolism	Chloroplast	Biotin carboxylase (ACCase complex)	56	EFVEICTDHGLEFIG	[47]
16	FAB2	Cre17.g701700	Metabolism	Chloroplast	Plastid acyl-ACP desaturase, d-9 stearate desaturase	45	DEGRHEIAYCKIMDG	[47]
17	GAP2	Cre07.g354200	Metabolism	Whole cell	Glyceraldehyde 3-phosphate dehydrogenase	40	DLVSTDFQGDNRSSIFDAKAGI	[48]
18	MPC1	Cre03.g172300	Metabolism	Integral component of membrane	Mitochondrial phosphate carrier protein	37	DVAKKTDSTKYAMP	
19	HSP70A	Cre08.g372100	HSP	Whole cell	Heat shock protein 70A	71	EQVFSTYSDNQPGV/Expressed protein (N-terminal)	[19]
20	HSP90B	Cre17.g730950	HSP	Whole cell	Heat shock protein 90B	87	GKDSKLKDLKESFK	

## Supplementary Materials

Supplementary File 1
