# Supplementary figures and images for: Identification of Reference and Biomarker Proteins in Chlamydomonas reinhardtii Cultured under Different Stress Conditions
Source: Int J Mol Sci. 2017 Aug 22;18(8):1822. doi: 10.3390/ijms18081822 (PMC5578208; doi:10.3390/ijms18081822)

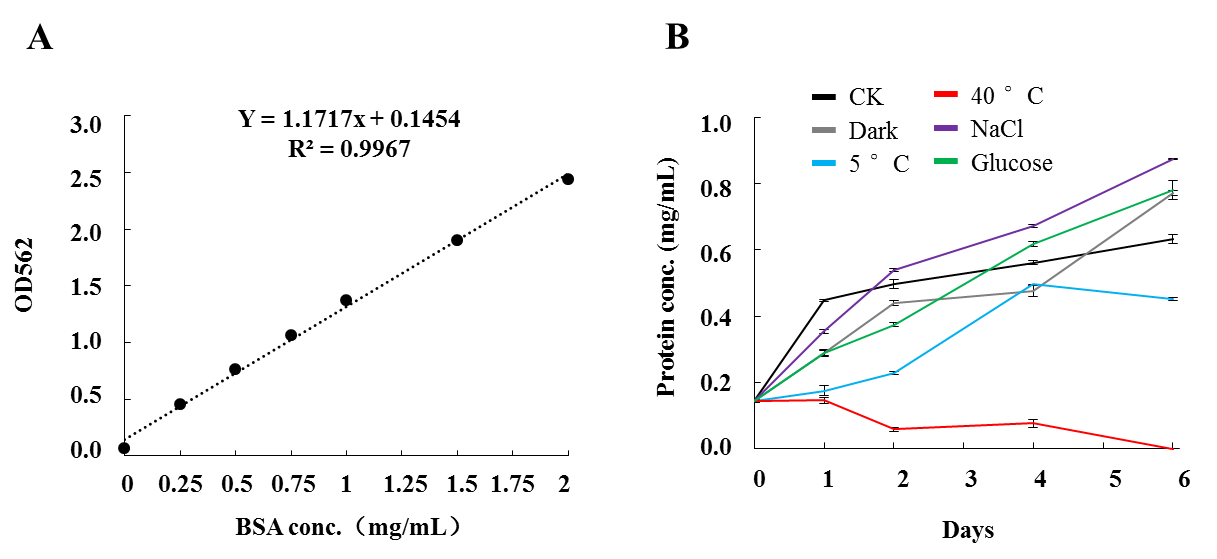

Supplement: Supplementary File 1 [file ijms-18-01822-s001.zip › S-Figure 1.tif]

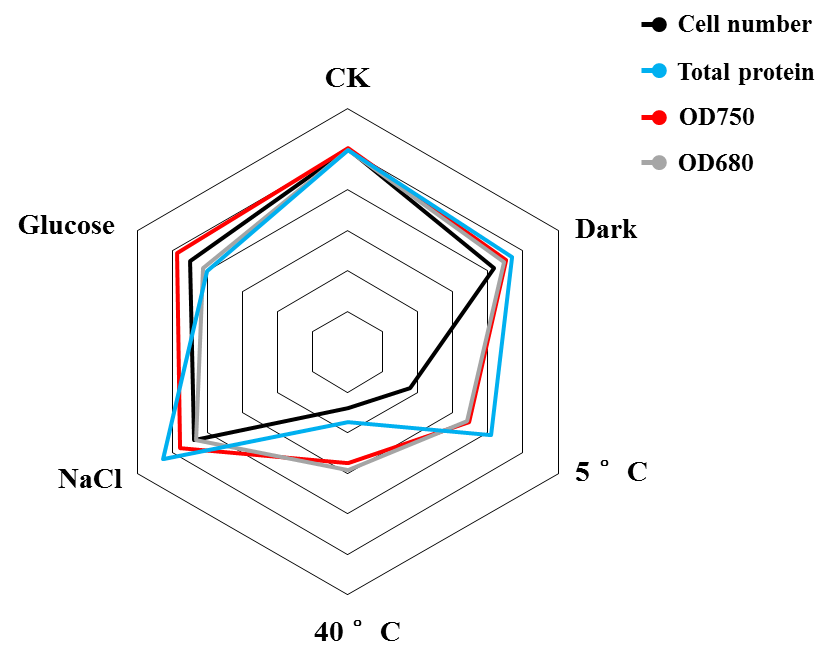

Supplement: Supplementary File 1 [file ijms-18-01822-s001.zip › S-Figure 2.tif]

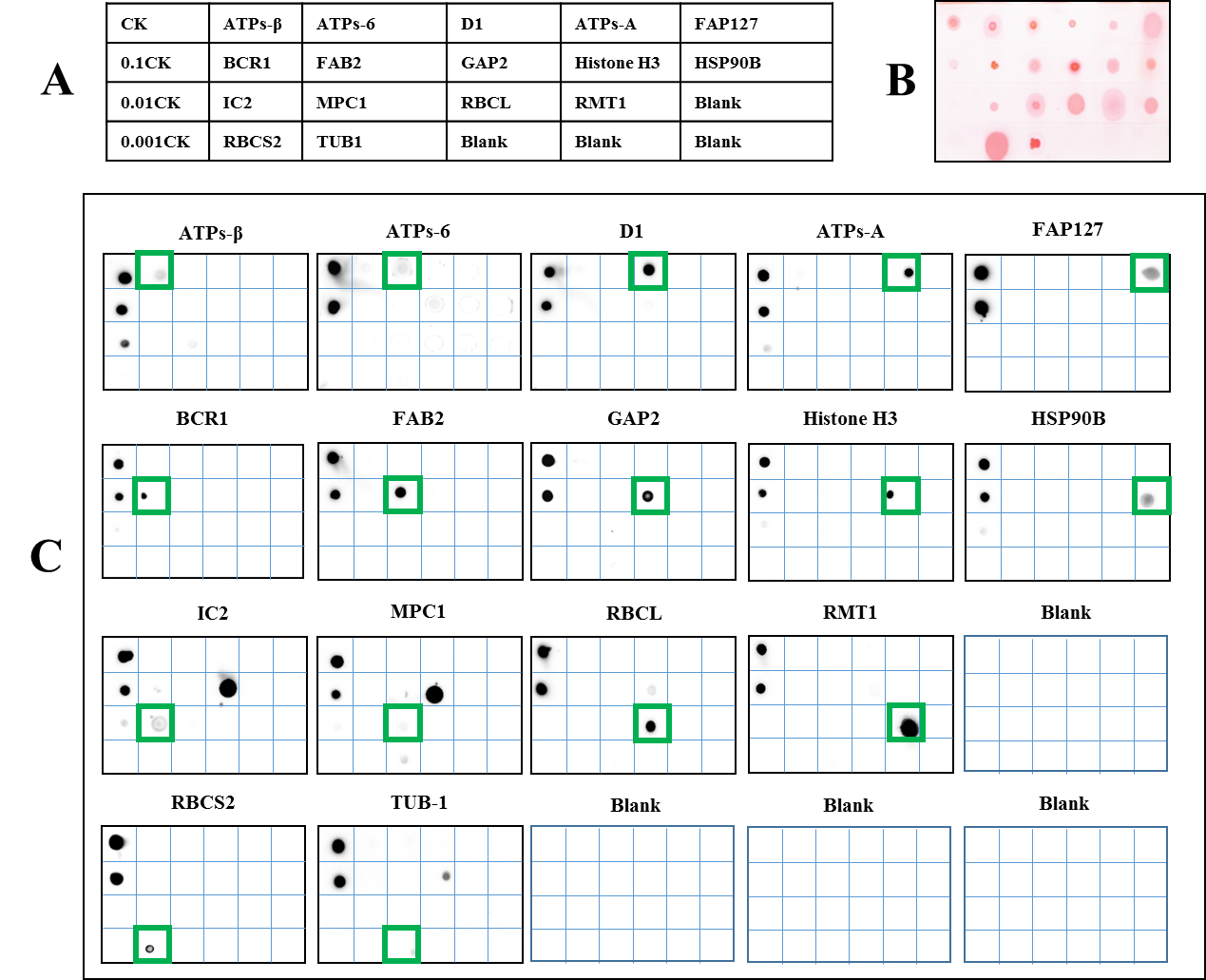

Supplement: Supplementary File 1 [file ijms-18-01822-s001.zip › S-Figure 3.tif]

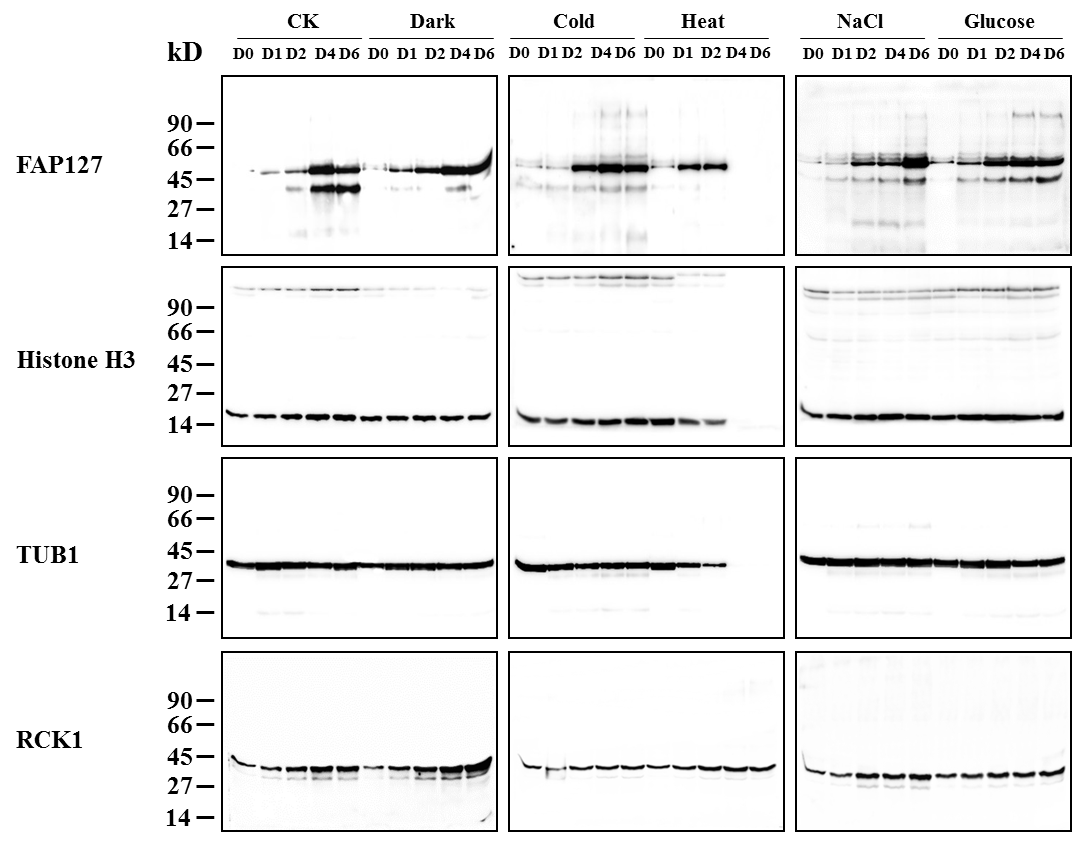

Supplement: Supplementary File 1 [file ijms-18-01822-s001.zip › S-Figure 4A.tif]

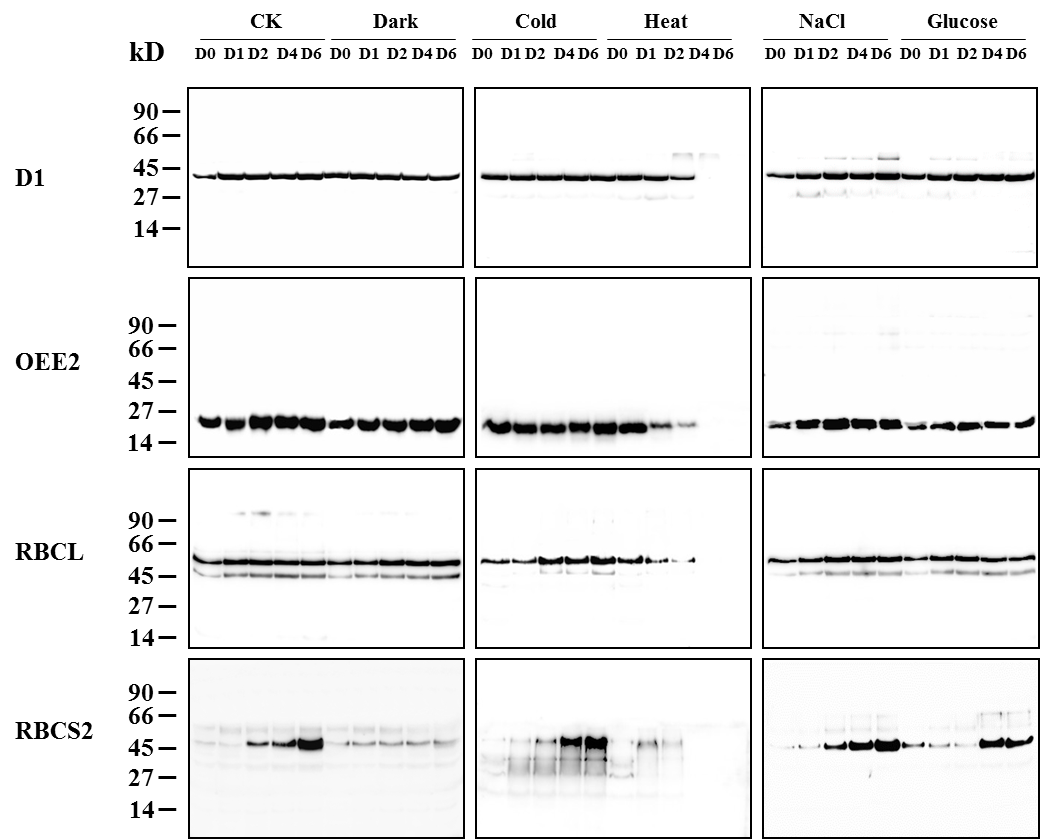

Supplement: Supplementary File 1 [file ijms-18-01822-s001.zip › S-Figure 4B.tif]

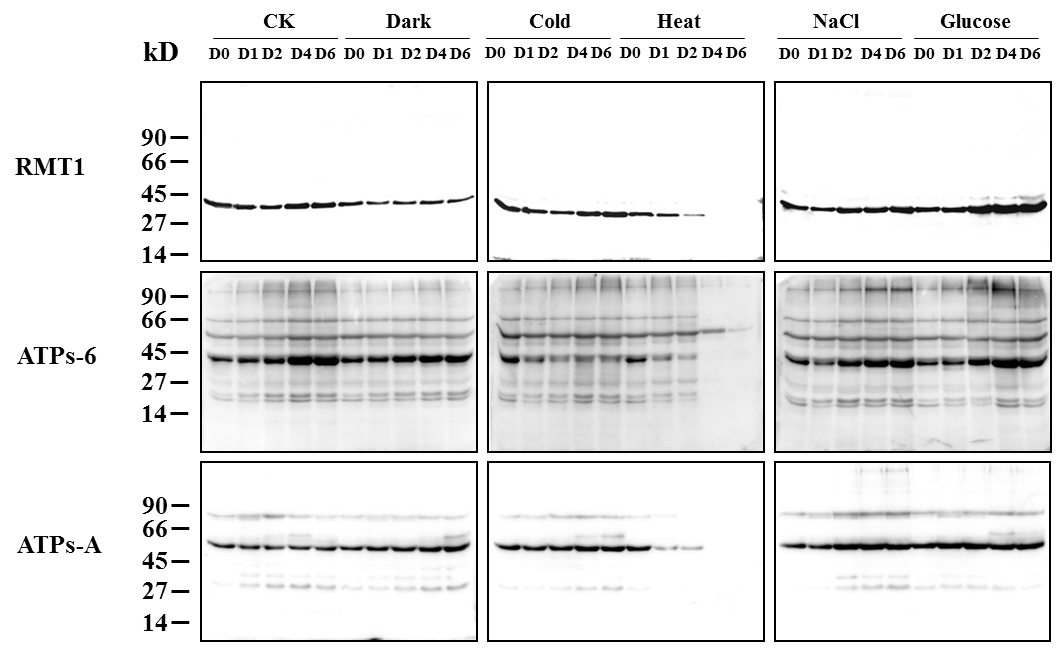

Supplement: Supplementary File 1 [file ijms-18-01822-s001.zip › S-Figure 4C.tif]

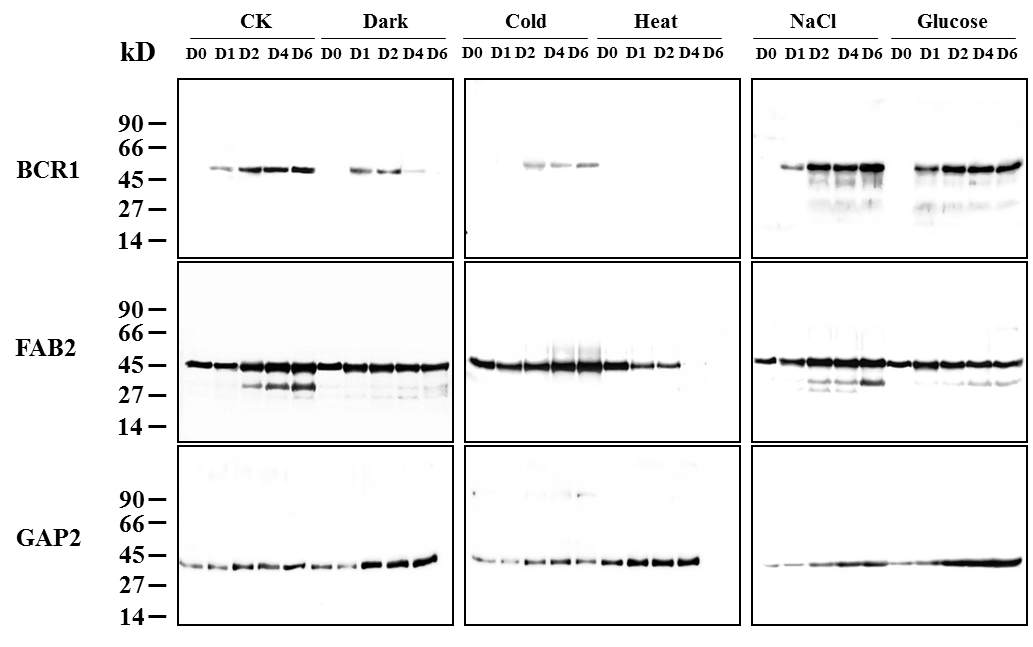

Supplement: Supplementary File 1 [file ijms-18-01822-s001.zip › S-Figure 4D.tif]

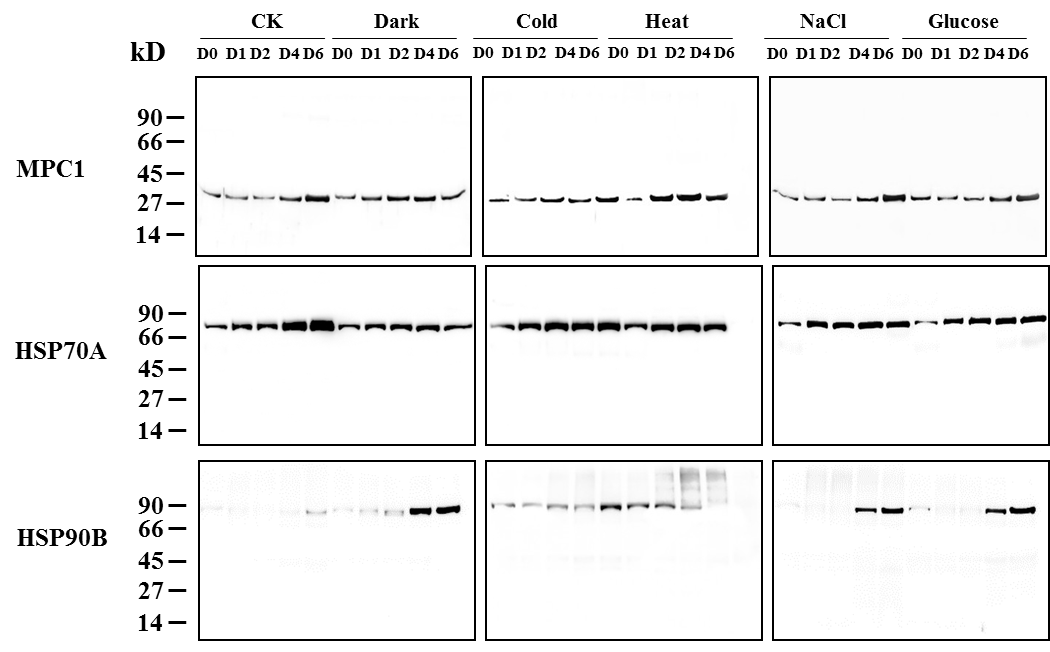

Supplement: Supplementary File 1 [file ijms-18-01822-s001.zip › S-Figure 4E.tif]

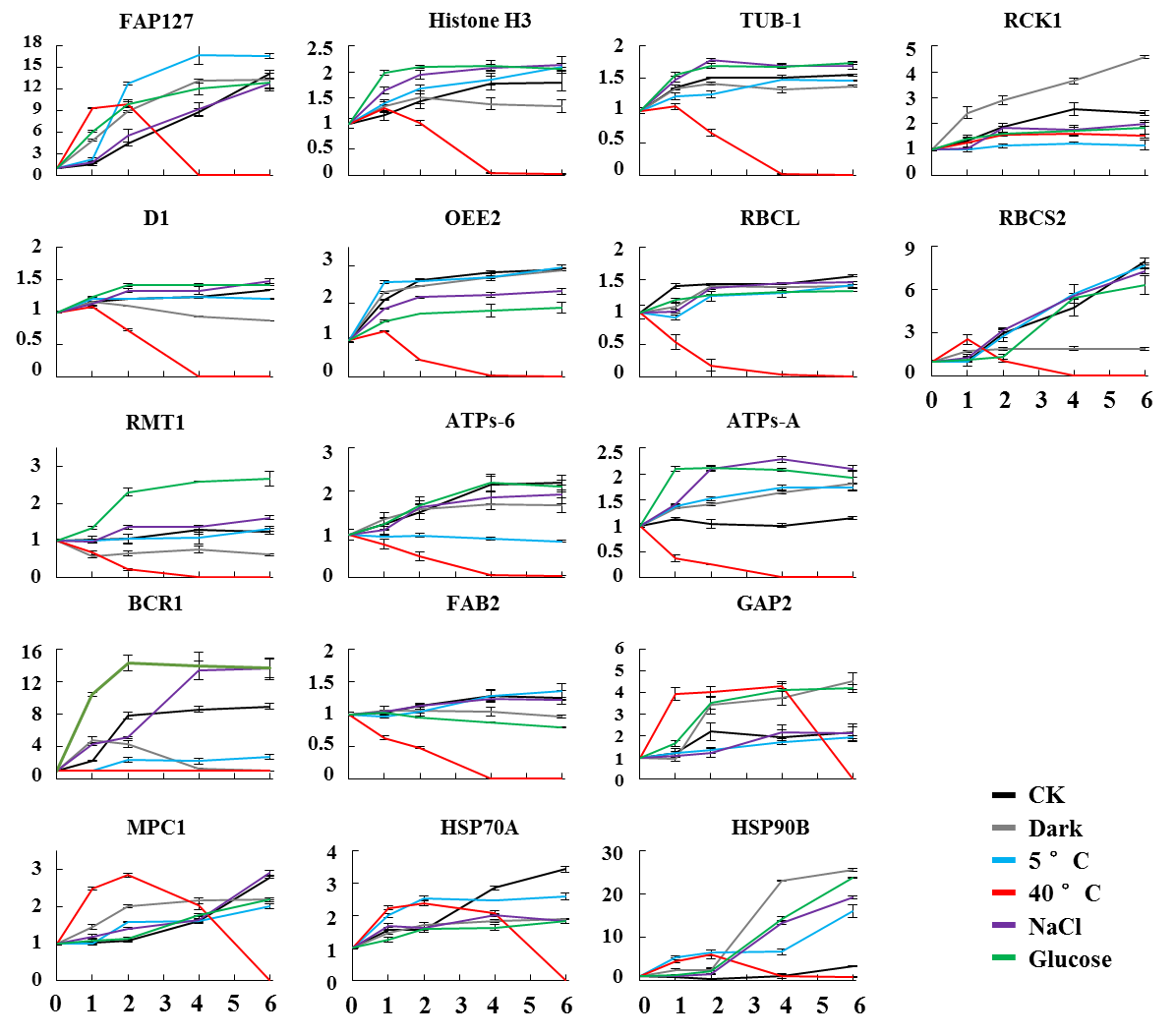

Supplement: Supplementary File 1 [file ijms-18-01822-s001.zip › S-Figure 5.tif]

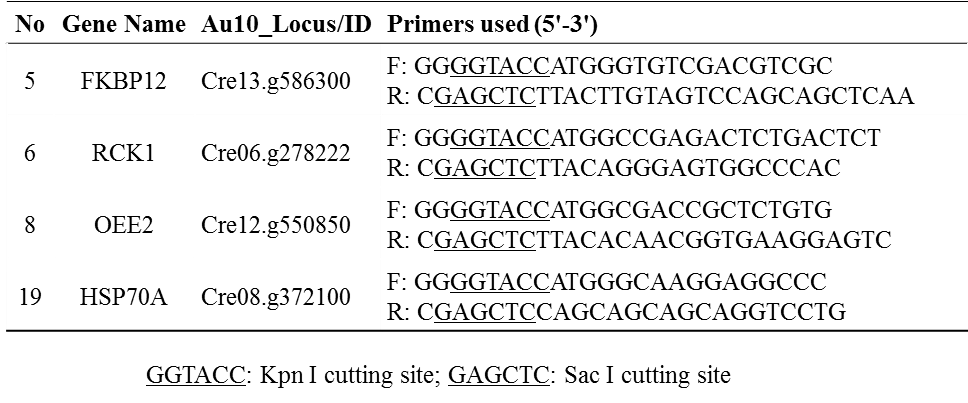

Supplement: Supplementary File 1 [file ijms-18-01822-s001.zip › S-Table 1.tif]
